# Supplementary material for: The interactome of intact mitochondria by cross-linking mass spectrometry provides evidence for coexisting respiratory supercomplexes
Source: Mol Cell Proteomics. 2017 Dec 8;17(2):216–32. doi: 10.1074/mcp.RA117.000470 (PMC5795388; doi:10.1074/mcp.RA117.000470)
Supplement: Supplemental Data [file supp_RA117.000470_133922_0_supp_23978_fzffwf.zip › spectra_annotation/mito_spectra_annotation/368-1-13-1-22-1.pdf]

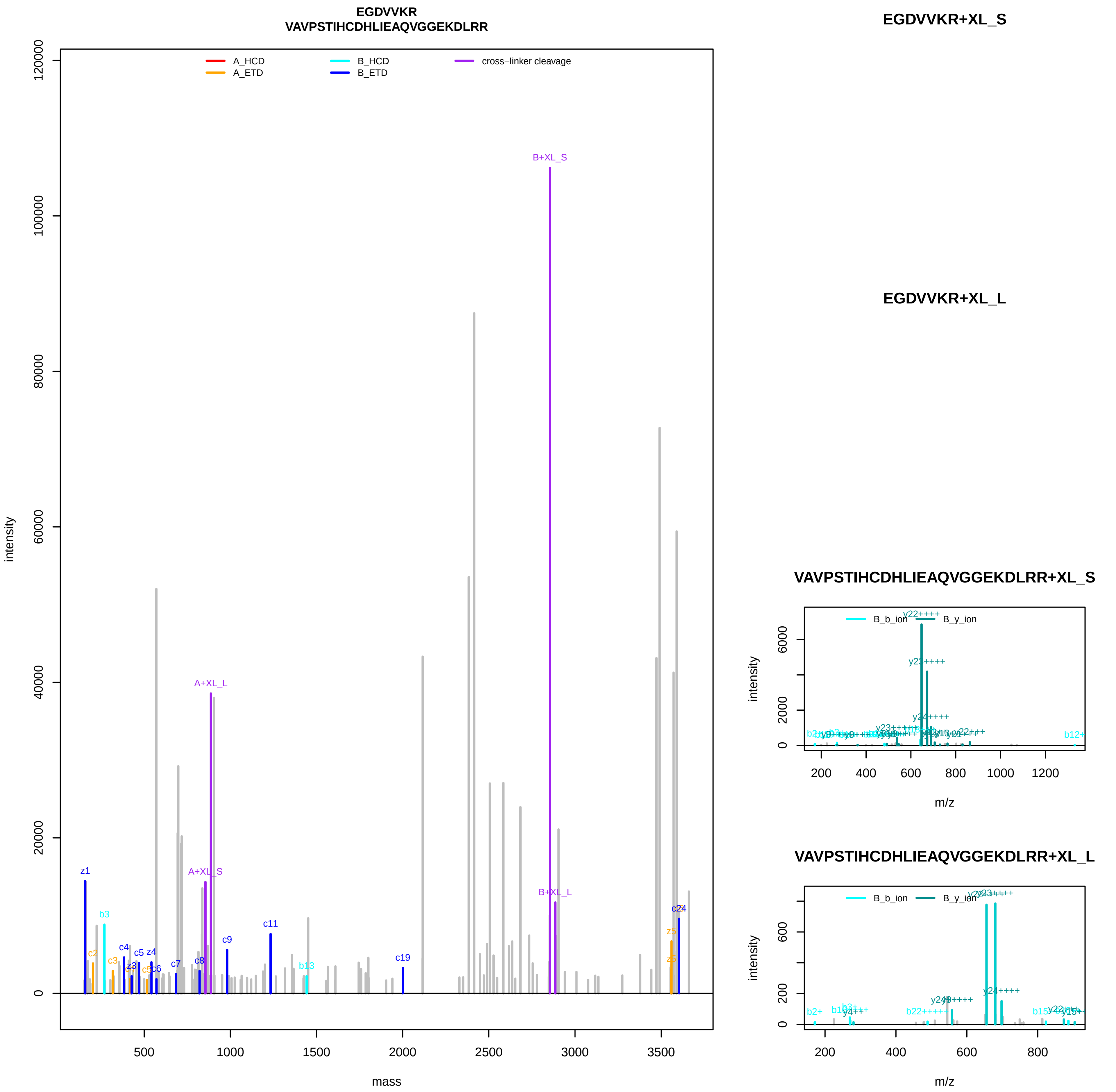
EGDVVKR
VAVPSTIHCDHLIEAQVGGEKDLRR
A_HCD
A_ETD
B_HCD
B_ETD
cross−linker cleavage
intensity
mass
B+XL_S
A+XL_L
A+XL_S
B+XL_L
z1
c2
b3
c3
c4
z3
c5
z4
c6
c7
c8
c9
c11
b13
c19
z5
c24
EGDVVKR+XL_S
EGDVVKR+XL_L
VAVPSTIHCDHLIEAQVGGEKDLRR+XL_S
B_b_ion
B_y_ion
y22++++
y23++++
y24++++
b12+
m/z
VAVPSTIHCDHLIEAQVGGEKDLRR+XL_L
b2+
y4++
b22+++++
y24++++
b15++
y15++
